# Myeloid decidual dendritic cells and immunoregulation of pregnancy: defective responsiveness to *Coxiella burnetii* and *Brucella abortus*

**DOI:** 10.3389/fcimb.2014.00179

**Published:** 2014-12-23

**Authors:** Laurent Gorvel, Amira Ben Amara, Mignane B. Ka, Julien Textoris, Jean-Pierre Gorvel, Jean-Louis Mege

**Affiliations:** ^1^CNRS UMR 7278, IRD198, INSERM U1095, Unite de Recherche sur les Maladies Infectieuses Tropicales Emergentes (URMITE), Aix-Marseille UniversityMarseille, France; ^2^UM2, INSERM U1104, CNRS, UMR7280, Centre d'Immunologie de Marseille Luminy, Aix-Marseille UniversityMarseille, France

**Keywords:** placenta, dendritic cell, phenotype, microarray, immunoregulation

## Abstract

Dendritic cells (DCs) are a component of the placental immune system, but their role in pregnancy is still poorly understood. Decidual DCs (dDCs) were selected from at-term pregnancy on the basis of CD14 and CD11c expression. A phenotypic analysis revealed that dDCs are characterized by the expression of monocyte-derived DC (moDCs) markers and specific markers such as HLA-G and its ligand ILT4. As demonstrated by whole-genome microarray, dDCs expressed a specific gene program markedly distinct from that of moDCs; it included estrogen- and progesterone-regulated genes and genes encoding immunoregulatory cytokines, which is consistent with the context of foeto-maternal tolerance. A functional analysis of dDCs showed that they were unable to mature in response to bacterial ligands such as lipopolysaccharide or peptidoglycan, as assessed by the expression of HLA-DR, CD80, CD83, and CD86. When dDCs were incubated with bacteria known for their placenta tropism, *Coxiella burnetii* and *Brucella abortus*, they were also unable to mature and to produce inflammatory cytokines. It is likely that the defective maturation of dDCs and their inability to produce inflammatory cytokines is related to the spontaneous release of IL-10 by these cells. Taken together, these results suggest that dDCs exhibit an immunoregulatory program, which may favor the pathogenicity of *C. burnetii* or *B. abortus*.

## Introduction

Dendritic cells (DCs) are sentinels that instruct the adaptive immune system at the interface of the host with environment. Following an encounter with microorganisms, they develop a maturation program associated with their migration to draining lymph nodes. The maturation program of DCs includes loss of endocytosis ability, dramatic changes in surface markers such as CD80, CD83, CD86, and membrane translocation of MHC class II molecules. Once mature, DCs are able to present the antigen to resting T cells (Banchereau and Steinman, 1998). The maturation program of DCs can be induced by microbial components such as lipopolysaccharide (LPS) and peptidoglycan (PGN) and modulated by the cytokine context. Hence, interferon (IFN)-γ or Tumor Necrosis Factor (TNF) drive inflammatory activation while interleukin (IL)-4, IL-10, or Transforming Growth Factor (TGF)-β induce an immunoregulatory response of DCs. This leads to Th1 or Th2 response, respectively (Akdis et al., 2012; Dzopalic et al., 2012). The functional properties of human DCs are dependent on DC location. Skin DCs are composed of epidermal Langerhans cells and different subtypes of dermal DCs that favor cell-mediated and antibody-mediated responses (Von Bubnoff et al., 2004; Kaplan et al., 2005; He et al., 2006). Intestinal DCs are essential to instruct the immune system about the presence of penetrating microorganisms but also to maintain tolerance toward commensals (Fleeton et al., 2004).

The placenta is a tissue dedicated to the exchange between mother and fetus and to feto-maternal tolerance. This latter relies on the presence of immune cells, mainly consisting of NK cells but also T lymphocytes, macrophages, and DCs (Erlebacher, 2013). While the role of NK cells and macrophages in feto-maternal tolerance is now being understood (Ben Amara et al., 2013; Erlebacher, 2013), the role of DCs remains unclear. DCs are present at the feto-maternal interface (Tagliani and Erlebacher, 2011), cycling endometrium and decidua. Their number is relatively low as compared to placenta macrophages (Erlebacher, 2013). The placenta-associated DCs are heterogeneous. Further, it has been described that decidua may contain mature DCs expressing CD83, which are found in clusters with CD3 T cells (Kämmerer et al., 2000), and immature DCs expressing CD14 and DC-SIGN (dendritic cell specific ICAM-grabbing non integrin, CD209) (Kämmerer et al., 2003). Decidual DCs (dDCs) play both antigen-presenting role and immunoregulatory role (Miyazaki et al., 2003; Blois et al., 2007; Gregori et al., 2010; Amodio et al., 2013).

In this report, we isolated and characterized dDCs from at-term placentas. They expressed classical phenotypic DC markers and also HLA-G and ILT4. The dDCs were characterized by a gene program in which estrogen and progesterone-regulated genes and genes encoding immunoregulatory cytokines were enriched. These DCs were unable to mature in response to bacteria-derived ligands such as LPS or PGN, and to bacteria known for their placenta tropism such as *Coxiella burnetii* and *Brucella abortus*. The spontaneous secretion of IL-10 combined with the defective production of inflammatory cytokines likely accounts for the immunoregulatory profile of dDCs. These results suggest that dDCs play an immunoregulatory role in feto-maternal tolerance, which is not broken down by *C. burnetii* and *B. abortus* and may contribute to their pathogenicity.

## Materials and methods

### Preparation of placental cells

Fifteen at-term placentas obtained by vaginal delivery were collected in the Gynecology-Obstetrics Department of the Hôpital de la Conception (Marseille, France) after written informed consent of healthy pregnant women. The study was approved by the Ethics Committee from Aix-Marseille University (N° 08-012). The placenta samples (approximately 150 g) were incubated in a solution consisting of Hank's Balanced Salt Solution (HBSS, Invitrogen, Cergy Pontoise, France), MgSO_4_, DNase I (Sigma-Aldrich, Saint-Quentin Fallavier, France) and 2.5% trypsin (Invitrogen) buffered with HEPES for 45 min and were then incubated for 30 min under gentle agitation at 37°C, as described previously (Ben Amara et al., 2013). The digestion products were then filtered through 100-μm pores, incubated in 50-ml tubes containing 2 ml fetal calf serum (FCS) and centrifuged at 1000× *g* for 15 min. The cells were counted, deposited on a Ficoll cushion and centrifuged at 700× *g* for 20 min. Mononuclear cells were recovered, and macrophages were discarded using magnetic beads coated with anti-CD14 Abs (Miltenyi Biotech, Paris, France). CD14^−^ cells were recovered and CD11c^+^ cells were sorted using magnetic beads (Miltenyi Biotec) coupled with anti-CD11c antibodies (Abs, Beckman Coulter, Villepinte, France). The purity of CD11c^+^ cells was higher than 95%.

Trophoblasts were isolated as previously described (Salcedo et al., 2013) with slight modifications. Briefly, isolated cells from placental samples were deposited on 25 and 60% Percoll (Sigma-Aldrich) phases and centrifuged at 1200× *g* for 20 min. Trophoblasts were isolated using anti-epidermal growth factor R (EGFR) Abs (Santa Cruz, Heidelberg, Germany) coupled to magnetic beads (Miltenyi Biotech). The purity of isolated trophoblasts was checked by flow cytometry using EGFR Abs and was higher than 96%. Trophoblasts were cultured in DMEM-F12 containing 10% FCS and antibiotics. Cell supernatants were collected 2 days after confluence and stored at −20°C.

### Preparation of moDCs

Blood from healthy donors was provided by the Etablissement Français du Sang (Marseille, France). Peripheral blood mononuclear cells (PBMCs) from buffy coats were recovered from the Ficoll-Hypaque interface after a 700× *g* centrifugation for 20 min. Monocytes were isolated from PBMCs using magnetic beads coupled with Abs specific for CD14, as previously described (Gorvel et al., 2014). Monocyte purity was higher than 98%. To obtain moDCs, monocytes were incubated in RPMI 1640 containing 20 mM HEPES, 2 mM glutamine, 10% FCS, 1 ng/ml IL-4, and 1 ng/ml granulocyte macrophage colony-stimulating factor (R&D Systems, Lille, France) for 7 days. The purity of moDCs was assessed by the absence of CD14 and the presence of CD11c, and purity was higher than 98%.

### Stimulation of moDCs and dDCs

moDCs and dDCs (2 × 10^5^ cells per assay) were stimulated with *Escherichia coli* LPS (Sigma-Aldrich, 100 ng/ml) and PGN (Sigma-Aldrich, 1 μg/ml) for 18 h. They were also incubated with *C. burnetii* (MOI 20:1) and *B. abortus* (MOI 20:1) for 18 h. *C. burnetii* organisms (RSA493 Nile Mile strain) were obtained by culture in L929 cells, as previously described (Barry et al., 2012). *B. abortus* strain 2308 was grown on tryptic soy agar (Sigma-Aldrich) at 37°C for 4–5 days, as previously described (Pizarro-Cerdá et al., 1998).

### Fluorescence microscopy

The moDCs and dDCs (10^5^ cells per assay) were cultured on glass slides for 18 h. After fixation in 3% paraformaldehyde for 15 min, they were permeabilized by 0.1% TritonX-100 for 2 min and then incubated for 30 min with bodipy phallacidin (Invitrogen) to label filamentous actin (F-actin). Cell nuclei were labeled with DAPI (Invitrogen) for 10 min and slides were mounted on Mowiol (Invitrogen). Pictures were taken using a confocal microscope DMI16000 (Leica, Nanterre, France) and analyzed using Image J software (National Institute of Health, USA).

In some experiments, moDCs and dDCs were incubated with *C. burnetii* and *B. abortus* for 18 h. *C. burnetii* and *B. abortus* organisms were revealed using human and bovine specific Abs, respectively. Secondary Abs consisted of anti-human and -bovine Abs coupled with 555 Alexa fluor. Pictures were taken using a confocal microscope DMI16000 (Leica, Nanterre, France) and merged using Image J software (National Institute of Health, USA). Superposition of red and green labeling induced yellow color on the picture.

### Flow cytometry

The moDCs and dDCs (10^5^ cells per assay) were incubated with HLA-DR and CD11c Abs (Beckman Coulter) in 400 μl of PBS containing 2% BSA for 30 min at 4°C. moDCs and plaDCs were then incubated with DC-SIGN, ASGPR, MARCO, Dectin-1, HLA-ABC, CD80, CD83, CD86, HLA-G, ILT4, BDCA-1 mAbs or isotypic controls (Beckman Coulter, Villepinte, France) for 30 min. They were then labeled with aquadead Amcyan (CellTrace) to exclude dead cells, as recommended by the manufacturer. After centrifugation, moDCs and dDCs were fixed in 3% paraformaldehyde for 15 min, washed in phosphate-buffered saline and analyzed using a Canto II flow cytometer associated with the software FACS Diva (Becton Dickinson, Pont de Claix, France). The dDCs were gated according CD11c expression and approximately 10,000 events were numerated. The results are given in percentage of positive cells.

### Microarrays

Total RNA of moDCs and dDCs (2 × 10^6^ cells per well) was extracted using the RNeasy minikit (Qiagen, Courtaboeuf, France) and DNAse treatment (Gorvel et al., 2014). The expression of modulated genes was analyzed using 4X44k Human Whole Genome microarrays (Agilent Technologies, Les Ulis, France) and three biological replicates per experimental condition, as recently described (Ben Amara et al., 2010). Sample labeling and hybridization were performed using One-Color Microarray-Based Gene Expression Analysis. Slides were scanned at a 5 μm resolution with a G2505C DNA microarray scanner (Agilent Technologies, Les Ulis, France). Image analysis and intra-array signal corrections were performed using Agilent Feature Extractor Software A.9.1.3.

Microarray data analysis was performed using the R (v.2.15) and Bioconductor libraries, as recently described (Moal et al., 2013). In brief, raw data were filtered and normalized using the Agi4x44PreProcess library. Unsupervised and supervised analyses were carried out using hierarchical clustering, principal component analysis (PCA) (made4 library, Culhane et al., 2005), and Significance Analysis of Microarray (SAM) algorithm (siggenes library). Genes were considered to be differentially expressed when absolute fold change (FC) was above 2.0. Functional enrichment analysis was performed on selected genes with DAVID tools (Dennis et al., 2003), using the Gene Ontology (GO) (Ashburner et al., 2000), and Kyoto Encyclopedia of Genes and Genomes (KEGG) (Okuda et al., 2008) pathways. Functional pathways were designed using the Cytoscape and Inkscape softwares.

### Western blotting

The moDCs and dDCs (10^6^ cells per assay) were scrapped in ice-cold RIPA buffer, as previously described (Barry et al., 2012). Proteins were separated by electrophoresis (40 μg of loaded proteins) and transferred onto nitrocellulose membranes (Amersham, Courtaboeuf, France). The membranes were probed with mouse Abs directed against chorionic somatomammotropin hormone 1 (CSH-1), also known as placental lactogen hormone, or α-tubulin (R&D Systems) for 18 h. The blots were incubated with horseradish peroxidase-conjugated Abs directed against mouse IgG (Pierce, Rockford, IL, USA). Bound Abs were detected using Immobilon Western Chemiluminescent HRP substrate (Millipore). The expression of CSH-1 was quantified by densitometric scanning and was normalized against α-tubulin. The results are expressed as relative intensities.

### Immunoassays

Supernatants from stimulated moDCs and dDCs were collected and freezed at −80°C. The concentrations of released cytokines were assessed using commercial ELISA kits. The sensitivity of IL-10 and IL-12p70 kits (R&D Systems) is 3.9 pg/ml and 2.5 pg/ml, respectively, and that of IL-6 kit (Becton Dickinson) is 4 pg/ml. The intra- and inter-variability of kits was less than 10%.

### Statistical analysis

The results are expressed as the means ± SD and were compared using the non-parametric Mann–Whitney *U*-test. When the comparisons involved more than two conditions, the analysis was made with ANOVA test. *P*-values less than 0.05 were considered significant.

## Results

### Phenotypic characterization of dDCs

**Figure 1:**
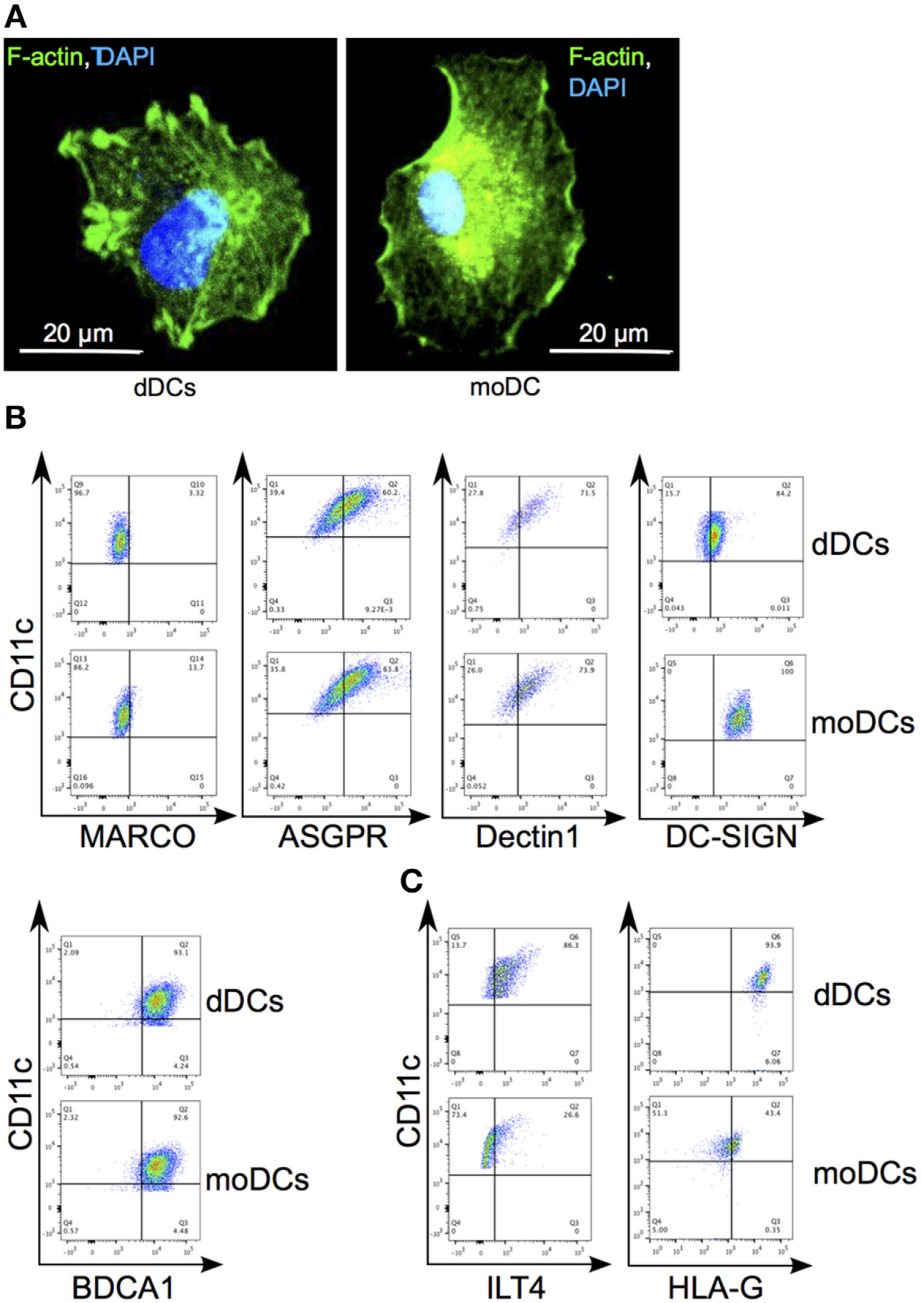
**Phenotypic characterization of dDCs. (A)** dDCs and moDCs were labeled with bodipy phallacidin (in green) and DAPI (in blue). One representative DC is shown. **(B)** The expression by dDCs of canonical molecules of DCs was assessed by flow cytometry. Dot-plots represent the expression of MARCO, ASGPR, DC-SIGN, and Dectin1 by CD11c^+^ cells. Quadrant gate represent the limit set by isotype control analysis. **(C)** The expression of HLA-G and ILT4 by plaDCs and moDCs by CD11c^+^ cells was assessed by flow cytometry. Quadrant gates represent the limit set by isotype control analysis. Dot-plots are representative of five different placentas.

We isolated myeloid DCs from decidual mononuclear cells by combining negative selection with anti-CD14 Abs and positive selection with anti-CD11c Abs. The morphology of the resulting CD14^−^CD11c^+^ DC subset, called dDCs, was similar to that of moDCs, another type of myeloid DCs, using bodipy phallacidin and confocal microscopy (Figure 1A). We then analyzed dDC expression of classical myeloid DC markers, including MARCO (macrophage R with collagenous structure), ASGPR (ascialoglycoprotein receptor), Dectin-1, DC-SIGN and BDCA-1 using flow cytometry. Both dDCs and moDCs were positive for CD11c. Less than 15% of dDCs and moDCs expressed membrane MARCO; more than 50% of dDCs and moDCs expressed BDCA-1, ASGPR and Dectin-1; DC-SIGN was largely expressed by dDCs and moDCs (Figure 1B). In contrast, dDCs strongly expressed HLA-G and its interacting molecule ILT4, which were poorly and not expressed by moDCs, respectively (Figure 1C). Taken together, these results show that dDCs differed from moDCs on the basis of HLA-G and ILT4 expression.

### Transcriptional analysis of dDCs

**Figure 2:**
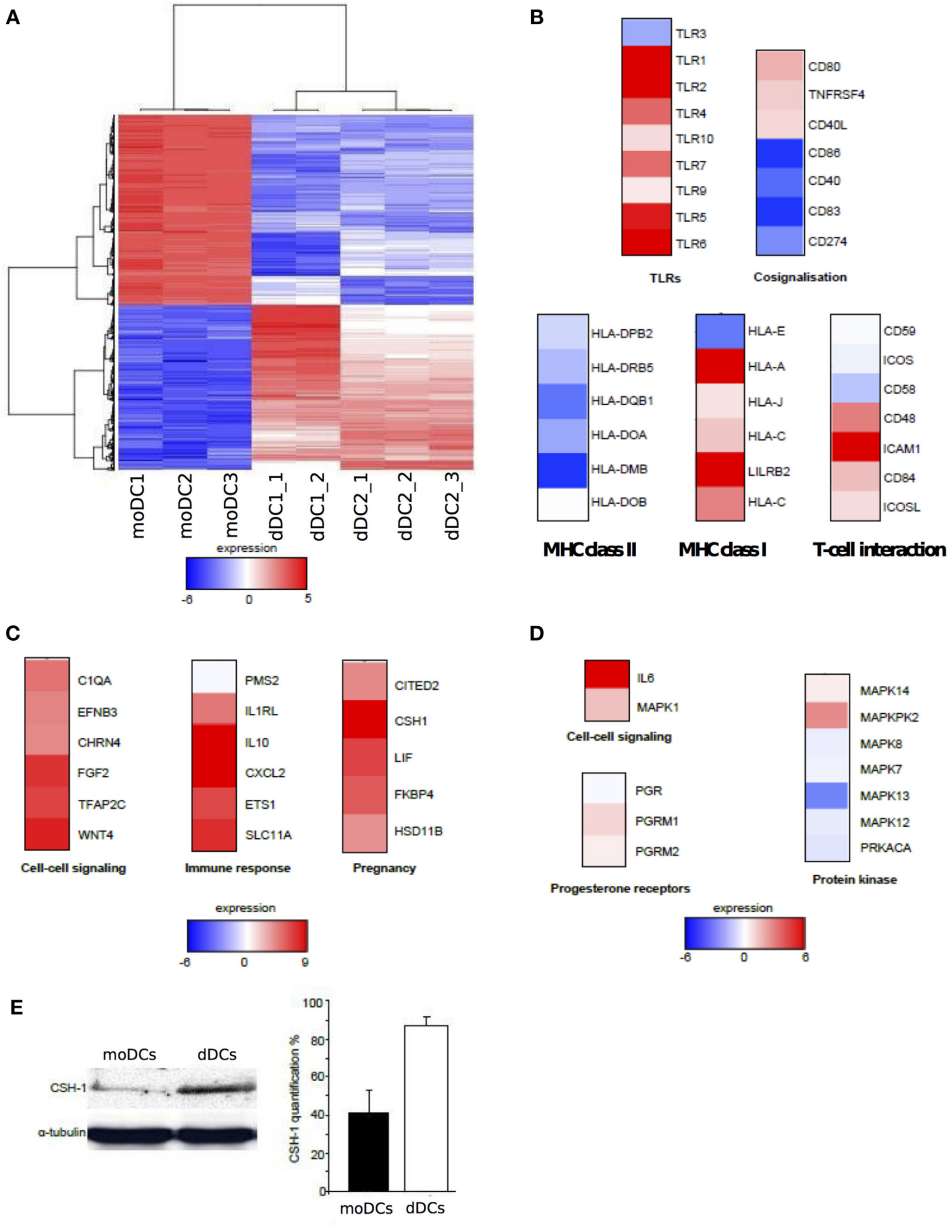
**Transcriptional analysis of dDCs. (A–D)** dDCs and moDCs were recovered, and microarray analysis was performed after RNA extraction. Only genes modulated in dDCs compared with moDCs were retained and the heatmap represents up-modulated (in red) and down-modulated (in blue) expression of genes **(A)** The functional annotation of the genes related to DC markers and those modulated in dDCs compared to moDCs is presented **(B)** Estrogen- and progesterone-modulated genes (**C** and **D**, respectively) were analyzed in dDCs compared with moDCs and functional annotations are shown. **(E)** The production of CSH-1 by dDCs and moDCs was assessed by western blot. The quantification was performed by densitometric scanning after normalization with α-tubulin. The results are representative of three different placentas.

As dDCs were phenotypically close to moDCs with the exception of the specific expression of HLA-G and ILT-4, we wondered if the analysis of gene expression would reveal specific transcriptional features. We found that 1525 genes were significantly modulated in dDCs compared with moDCs (using a *FC* > 2.0 and a False Discovery Rate (FDR) < 0.01) (Figure 2A). They consisted of 672 up-modulated genes and 853 down-modulated genes. We then selected clusters of genes involved in DC phenotype and functions, namely pathogen recognition (TLRs), co-signalisation and antigen presentation (MHC class I and II molecules, T-cell interaction), and investigated their transcriptional expression (Figure 2B). The expression of the genes encoding TLR1, TLR2, TLR4, TLR5, TLR6, and TLR7 was up-modulated in dDCs, whereas the expression of TLR9 and TLR10 was similar in dDCs and moDCs. TLR3 was found to be slightly depressed in dDCs. Most of the genes involved in DC co-signalisation function, *CD86, CD40, CD83*, and *CD274* (also known as *PDL1*), and those encoding MHC class II molecules were strongly down-modulated. In contrast, the majority of the genes encoding MHC class I molecules, and specifically *HLA-G* and its ligand *ILT4* (*LILRB2*) were up-modulated in dDCs. Finally, among the genes involved in the interaction with T-cells, *ICAM1* was highly up-modulated (Figure 2B). Taken together, these results showed that most of the genes involved in DC functions were down-modulated in dDCs compared with moDCs with the exception of *TLR* genes and genes encoding MHC class I molecules.

The second feature of transcriptional signature of dDCs is the impact of the placenta microenvironment. As estrogens and progesterone are major components of placenta microenvironment, we investigated estrogen and progesterone-regulated genes in dDCs compared with moDCs. We found that 17 genes known to be regulated by estrogens and 12 progesterone-regulated genes were modulated in dDCs. The 17 estrogen-regulated genes belong to the GO terms “cell–cell signaling,” “immune response” and “pregnancy”. These genes were up-modulated with the exception of PMS2 (Figure 2C). The 12 progesterone-modulated genes consisted of “cell–cell signaling,” “progesterone receptors and sub-units,” and “protein kinase” GO terms. These genes were poorly modulated at the exception of the *IL6* gene that was highly up-modulated (Figure 2D). The *CSH1* gene was highly expressed in dDCs and CSH-1 is strongly produced during pregnancy (Huddleston and Schust, 2004). We determined the presence of CSH-1 in dDCs and moDCs by immunoblotting. We found that dDCs, but not moDCs, constitutively produced CSH-1 (Figure 2E). These results suggested that the differences between dDCs and moDCs rely largely on their hormonal microenvironment, especially estrogens.

### Modulation of cytokine pathways in dDCs

**Figure 3:**
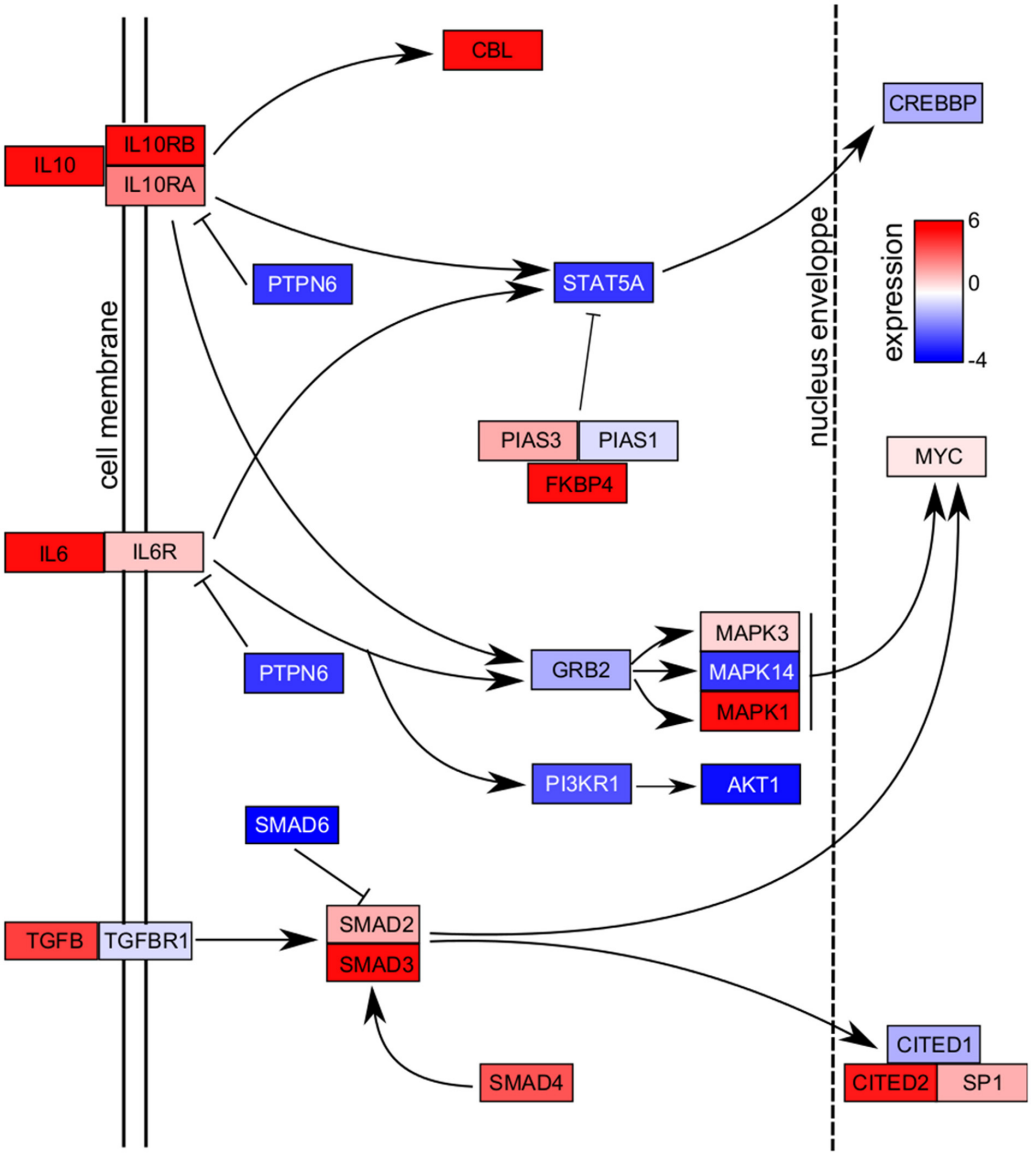
**Cytokine pathways**. The dDCs and moDCs were recovered, and microarray analysis was performed after RNA extraction. The genes modulated in dDCs compared with moDCs were retained and the *IL10, IL6*, and *TGFB* pathways are presented. Up-modulated molecules are shown in red and down-modulated molecules in blue. Abbreviations: AKT, v-akt murine thymoma viral oncogene homolog; CBL, E3 ubiquitin protein ligase; CITED, Cbp/p300-interacting transactivator, with Glu/Asp-rich carboxy-terminal domain; CREBBP, CREB-binding protein; FKBP4, F506-binding protein 4; GRB, growth factor R-bound protein; MYC, v-myc avian myelocytomatosis viral oncogene homolog; PIAS, protein inhibitor of activated STAT; PTPN6 protein tyrosine phosphatase non receptor type 6; SP, specificity protein; STAT5A, signal transducer and activator of transcription 5A.

Since dDCs exhibited a transcriptional program in which genes associated with DC maturation were essentially down-modulated, we wondered if signaling pathways were altered. We selected two pathways, IL-10 and TGF-β known for their role in fetal tolerance. We found that the expression of the genes encoding IL-10 and TGF-β was higher in dDCs than in moDCs (Figure 3). In the *IL10* pathway, the genes encoding IL-10R (*IL10RA* and *IL10RB*) were also up-modulated, but effector molecules such as *STAT5* and *CREBBP* were down-modulated. It is noteworthy that *STAT5* inhibitors (*PIAS3* and *FKBP4* genes) were up-modulated in dDCs. The *TGFβ* pathway was mainly up-modulated in dDCs, especially *SMAD* molecules. Only *SMAD6*, which is an inhibitor of the SMAD signaling cascade, was down-modulated. Finally, genes encoding nuclear transcription factors such as CITED2 (MSG related protein 1), MYC and SP1 were up-modulated whereas *CREBBP* and *CITED1* genes were down-modulated (Figure 3). Taken together, these results suggested that the *TGFβ* pathway was fully active in dDCs whereas the *IL10* pathway was partially activated.

### Maturation of dDCs and response to microbial ligands

**Figure 4:**
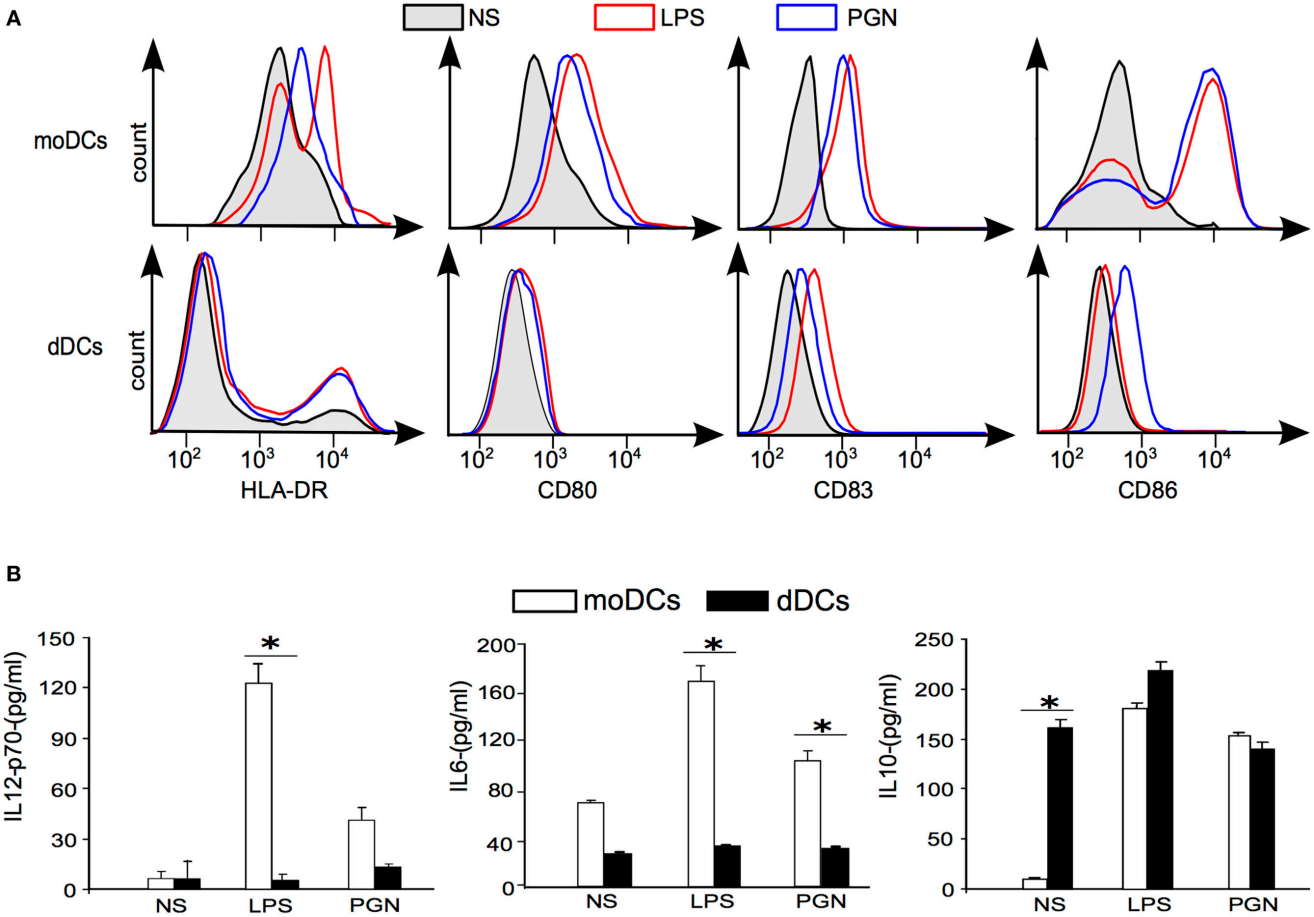
**plaDCs and TLR ligands. (A)** The expression of maturation markers was determined by flow cytometry in moDCs and dDCs stimulated with LPS or PGN for 18 h. Black lines and gray areas represent naive moDCs and dDCs; in red, LPS stimulation; in blue, PGN stimulation. **(B)** moDCs and dDCs were either stimulated or not by LPS or PGN for 18 h. Cytokine release was assessed by ELISA, and the results representative of five different experiments are expressed in pg/ml. “*” represent significative variations between conditions (+/− SD).

As the transcriptional signature of dDCs reflects an immunoregulatory profile, we investigated their ability to mature in the presence of LPS or PGN, known to induce the maturation of moDCs, as determined by the membrane expression of HLA-DR, CD80, CD83, and CD86. In the absence of stimulation, HLA-DR was strongly expressed by moDCs. In contrast, 90% of dDCs weakly expressed HLA-DR whereas only 8% of dDCs expressed HLA-DR at a high level. LPS and PGN increased the expression of HLA-DR in moDCs, but were unable to increase the membrane expression of HLA-DR in the majority of dDCs (Figure 4A). The expression of CD80, CD83, and CD86 was similar in unstimulated dDCs and moDCs. LPS and PGN markedly increased the expression of CD80, CD83, and CD86 in moDCs but were unable to substantially increase their expression in dDCs (Figure 4A). Hence, dDCs were unable to fully mature in response to TLR ligands. We then investigated the ability of dDCs to release inflammatory and immunoregulatory cytokines. The unstimulated production of IL-12p70 and IL-6 was similar in dDCs and moDCs. While LPS and PGN stimulated IL-12p70 and IL-6 release by moDCs, no effect was observed in dDCs (Figure 4B). The profile of IL-10 production was completely different. Indeed, unstimulated dDCs, but not moDCs, spontaneously released high levels of IL-10. The stimulation of dDCs by TLR ligands such as LPS and PGN did not affect the release of IL-10 by dDCs but markedly increased IL-10 release by moDCs (Figure 4B). Taken together, these results show that dDCs were unable to mature in response to ligands that induce the maturation of moDCs and to produce inflammatory cytokines in response to TLR ligands.

### Maturation of dDCs and response to intracellular bacteria

**Figure 5:**
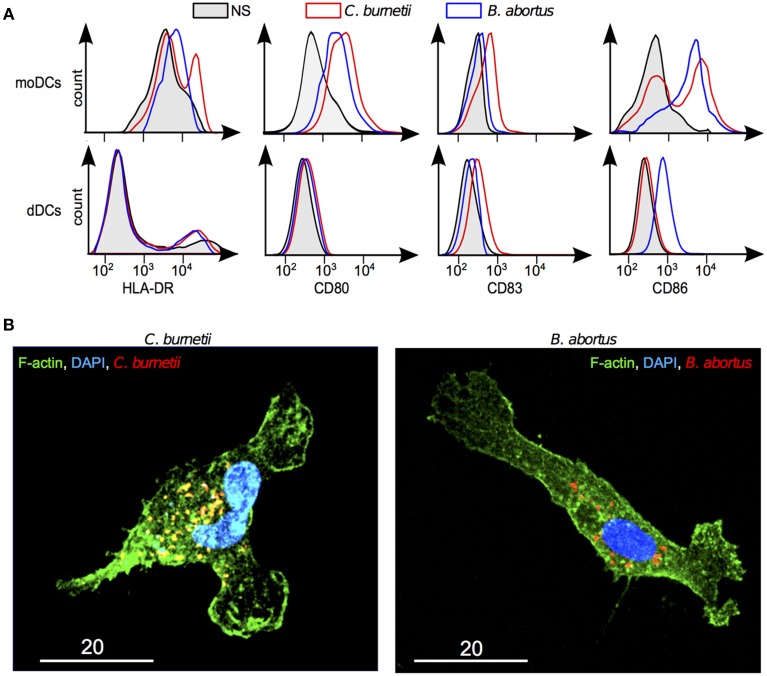
**Response of dDCs to *C. burnetii* and *B. abortus***. DDCs were incubated with *C. burnetii* or *B. abortus* for 18 h. **(A)** The expression of maturation markers was determined by flow cytometry in moDCs and dDCs stimulated or not by *C. burnetii* or *B. abortus*. In black and gray areas, naive moDCs and dDCs; in red, *C. burnetii* stimulation; in blue, *B. abortus* stimulation. The results are representative for five experiments. **(B)** After washing, bacteria were labeled with specific Abs, F-actin with bodipy phallacidin and nuclei with DAPI. dDCs were observed by confocal microscopy. The micrographs reveal bacteria (in red or yellow), F-actin (in green) and nuclei (in blue).

The inability of dDCs to mature in response to TLR ligands may create a favorable context for intracellular bacteria and specifically for the bacteria with a tropism for placenta such as *C. burnetii* and *B. abortus*. We tested the ability of *C. burnetii* and *B. abortus* to induce the membrane expression of DC maturation markers. The expression of HLA-DR, CD80, CD83, and CD86 was increased in *C. burnetii*-stimulated moDCs and that of CD80 and CD86 in *B. abortus*-stimulated moDCs. In contrast, *C. burnetii* did not affect the expression of HLA-DR, CD80, CD83, and CD86 by dDCs. The stimulation of dDCs by *B. abortus* remained silent at the exception of a weak increased expression of CD86 (Figure 5A). We verified that the lack of response to bacteria was not due to a lack of interaction. *C. burnetii* and *B. abortus* entered DC as shown in Figure 5B. These results showed that dDCs did not mature in response to *C. burnetii* and *B. abortus*, suggesting an intrinsic deficiency of dDC maturation.

## Discussion

In this paper, we characterized myeloid dDCs from third-trimester placentas. This DC population was isolated by negative selection through a CD14 column, followed by a positive selection through CD11c column. This procedure excluded placenta macrophages that highly express CD14 (Ben Amara et al., 2013) and also myeloid DCs that express CD14 and DC-SIGN (Erlebacher, 2013). The CD14^−^CD11c^+^ dDCs expressed BDCA-1, ASGPR, Dectin-1, and DC-SIGN. The expression of lectins such as ASGPR and Dectin-1 is characteristic of immature DCs. Indeed, IL-10-producing CD4^+^ T cells maintain DC in an immature status in which ASGPR is expressed (Kaisho and Akira, 2003; Li et al., 2012). DC-SIGN is expressed mainly by immature DCs and defective signaling through DC-SIGN may be involved in immune tolerance (Valladeau et al., 2001; Geijtenbeek et al., 2004). The CD11c^+^BDCA1^+^ dDCs are also present in the first trimester (Erlebacher, 2013), suggesting a stability of DC subsets during pregnancy.

We also provided evidence that dDCs were characterized by a specific transcriptional repertoire when compared with moDCs. They expressed TLR2 and TLR4, which are involved in the recognition of PGN and LPS, respectively (Kaisho and Akira, 2003), suggesting that dDCs recognize gram-negative and gram-positive bacteria. Interestingly, the genes encoding MHC class II molecules and associated pathways were down-modulated in dDCs compared with moDCs whereas the expression of genes encoding MHC class I molecules and associated pathways were mostly up-modulated. We can suppose that dDCs had a lower ability to process and present vacuolar antigens. The transcriptional signature of cytokines also evokes an immunoregulatory role for dDCs. Indeed, *IL6* and *IL10* genes were up-modulated in dDCs as compared with moDCs even if their downstream signaling effectors were down-modulated. The *TGFβ* pathway was clearly up-modulated in dDCs, suggesting that this pathway may play a role in fetal tolerance (Svajger et al., 2010; Erlebacher, 2013). The comparison of the transcriptional program of dDCs with that of decidual CD14^+^ macrophages and multinucleated giant cells (MGCs) revealed that dDCs were close to CD14^+^macrophages with which they exhibited a common placental signature. They were markedly distinct from moDCs and macrophages derived from monocytes or MGC (Supplementary Material).

As phenotypic and transcriptional features of dDCs suggested a tolerogenic signature, we tested the ability of dDCs to mature in response to TLR agonists known to induce the maturation of moDCs. A few proportions of dDCs expressed HLA-DR in contrast to moDCs upon stimulation with microbial ligands. In addition, these latter increased only marginally the membrane expression of CD83 and CD86 in dDCs. This was markedly different from first trimester CD83^+^ dDCs that express a mature DC phenotype (Kämmerer et al., 2000). We also found that dDCs were poorly inflammatory. Indeed, they did not release significant levels of IL-6 and IL-12p70 in response to LPS and PGN in contrast to moDCs. The dDCs spontaneously released high levels of IL-10, as previously found for trophoblasts, decidual macrophages, and uterine NK cells (Huddleston and Schust, 2004). As a consequence, LPS and PGN were unable to increase the release of IL-10 by dDCs while these agonists dramatically increased the release of IL-10 by moDCs. This demonstrates that dDCs were hyporeactive to inflammatory agonists. The tolerogenic signature of dDCs may prevent the development of immune response to intracellular bacteria with placenta tropism. Hence, *C. burnetii* and *B. abortus* were unable to induce their maturation This response was specific because *C. burnetii* or *B. abortus* were able to induce the maturation of moDCs. It has been also reported that dDCs locally present the antigen to decidual T cells in ways that minimize Th1 responses and reinforce the immunodepression associated with pregnancy, thus favoring the replication of pathogens within the placenta, such as *Listeria monocytogenes* (Abram et al., 2003), *C. burnetii* (Ben Amara et al., 2010) or *B. abortus* (Salcedo et al., 2013).

The mechanisms underlying dDC properties that favor the persistence of intracellular bacteria may involve other mechanisms than the lack of inflammatory cytokines. Indeed, dDCs expressed specific molecules such as HLA-G and its ligand ILT4. HLA-G is known for its immunosuppressive properties in normal and pathological conditions (Ristich et al., 2005). In the presence of soluble HLA-G tetramers, moDCs are not able to completely mature (McIntire and Hunt, 2005). The interaction of HLA-G with ILT4 limits trophoblast lysis by NK cells and increases the production of TGF-β and immunosuppressive cytokines in decidual macrophages and CD83^+^ DCs from first trimester (McIntire and Hunt, 2005). Sex hormones likely play a major role in the activity of placenta cells. It has been shown that progesterone increases the expression of HLA-G and induces a tolerogenic profile in uterine DCs (Szekeres-Bartho et al., 2009). Even if estrogens have been described as enhancers of DC maturation (Hughes and Clark, 2007; Nofer, 2012; Seillet et al., 2013), they have also been described as inhibiting DC maturation during RNA-virus infections (Escribese et al., 2008) and limiting the production of defensins by moDCs and myeloid DCs (Escribese et al., 2011). It is likely that estrogens are more potent than progesterone to affect the functional activity of dDCs, as suggested by microarray analysis. Among the estrogen-regulated genes, we found that *CSH1* gene and CSH-1 protein that is involved in prolactin secretion were highly up-modulated in dDCs. CSH-1 and prolactin participate to normal development of pregnancy because a decreased production of prolactin or CSH-1, as found in preeclampsia, an inflammatory disease of the placental tissue, results in retarded growth (Männik et al., 2012). We hypothesized that the hormonal context affected the ability of dDCs to mature and to produce inflammatory cytokines.

In this report, we found that dDCs exhibit specific features in addition to the markers of myeloid DCs. They were unable to mature and to produce inflammatory cytokines in response to agonists known to induce DC maturation and were strongly influenced by their tolerogenic hormonal microenvironment. These properties may contribute to the feto-maternal tolerance and to the pathogenicity of intracellular bacteria with placenta tropism, as dDCs were also unable to respond properly to such pathogens.

### Conflict of interest statement

The authors declare that the research was conducted in the absence of any commercial or financial relationships that could be construed as a potential conflict of interest.
