# Supplementary figures and images for: Myeloid decidual dendritic cells and immunoregulation of pregnancy: defective responsiveness to Coxiella burnetii and Brucella abortus
Source: Front Cell Infect Microbiol. 2014 Dec 23;4:179. doi: 10.3389/fcimb.2014.00179 (PMC4275036; doi:10.3389/fcimb.2014.00179)

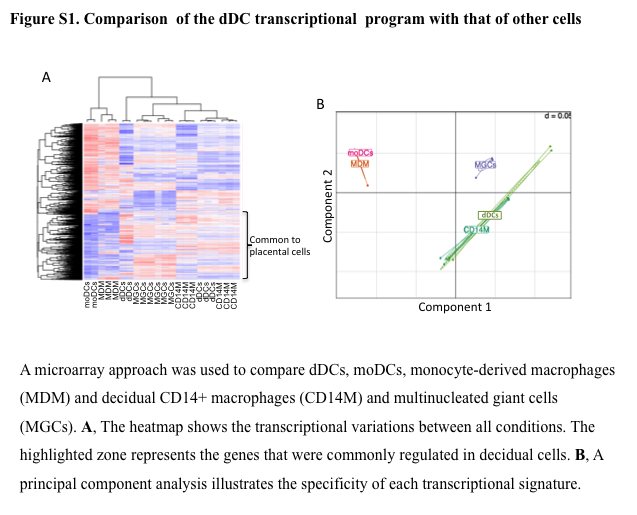

Supplement: Supplementary file 1 [file Image1.TIF]
